# Hif1α/Dhrs3a Pathway Participates in Lipid Droplet Accumulation via Retinol and Ppar-γ in Fish Hepatocytes

**DOI:** 10.3390/ijms241210236

**Published:** 2023-06-16

**Authors:** Jingjing Tian, Yihui Du, Binbin Wang, Mengmeng Ji, Hongyan Li, Yun Xia, Kai Zhang, Zhifei Li, Wenping Xie, Wangbao Gong, Ermeng Yu, Guangjun Wang, Jun Xie

**Affiliations:** 1 Key Laboratory of Aquatic Animal Immune Technology of Guangdong Province, Pearl River Fisheries Research Institute, Chinese Academy of Fishery Sciences, Guangzhou 510380, China; duyihui2021@163.com (Y.D.); wbb08220088@163.com (B.W.); jimengmengdongyi@163.com (M.J.); lihongyan@prfri.ac.cn (H.L.); xy@prfri.ac.cn (Y.X.); zk@prfri.ac.cn (K.Z.); lzf@prfri.ac.cn (Z.L.); xiewp@prfri.ac.cn (W.X.); gwb@prfri.ac.cn (W.G.); yem@prfri.ac.cn (E.Y.); gjwang@prfri.ac.cn (G.W.); 2 Key Laboratory of Tropical and Subtropical Fishery Resource Application and Cultivation, Pearl River Fisheries Research Institute, Chinese Academy of Fishery Sciences, Guangzhou 510380, China; 3 Hainan Fisheries Innovation Research Institute, Chinese Academy of Fishery Sciences, Sanya 572024, China

**Keywords:** aquaculture, fatty liver, fatty acid, hypoxia, cell culture, lipid droplet, lipoprotein, liver, retinoids

## Abstract

Excessive hepatic lipid accumulation is a common phenomenon in cultured fish; however, its underlying mechanisms are poorly understood. Lipid droplet (LD)-related proteins play vital roles in LD accumulation. Herein, using a zebrafish liver cell line (ZFL), we show that LD accumulation is accompanied by differential expression of seven LD-annotated genes, among which the expression of *dehydrogenase*/*reductase* (*SDR family*) *member 3 a*/*b* (*dhrs3a*/*b*) increased synchronously. RNAi-mediated knockdown of *dhrs3a* delayed LD accumulation and downregulated the mRNA expression of *peroxisome proliferator-activated receptor gamma* (*pparg*) in cells incubated with fatty acids. Notably, Dhrs3 catalyzed retinene to retinol, the content of which increased in LD-enriched cells. The addition of exogenous retinyl acetate maintained LD accumulation only in cells incubated in a lipid-rich medium. Correspondingly, exogenous retinyl acetate significantly increased *pparg* mRNA expression levels and altered the lipidome of the cells by increasing the phosphatidylcholine and triacylglycerol contents and decreasing the cardiolipin, phosphatidylinositol, and phosphatidylserine contents. Administration of LW6, an hypoxia-inducible factor 1α (HIF1α) inhibitor, reduced the size and number of LDs in ZFL cells and attenuated *hif1αa*, *hif1αb*, *dhrs3a*, and *pparg* mRNA expression levels. We propose that the Hif-1α/Dhrs3a pathway participates in LD accumulation in hepatocytes, which induces retinol formation and the Ppar-γ pathway.

## 1. Introduction

Currently, excessive accumulation of liver fat in cultured fish is a common phenomenon in the fish industry, representing a main risk factor for poor health status, high mortality, and decreased quality of fish fillets. This prevalent issue has become a bottleneck in the healthy development of the industry. In fish, fatty liver is mainly caused by the accumulation of lipid droplets (LDs) in hepatocytes. Thus, regulating the size and quantity of LDs is a key factor in controlling fatty liver disease. LDs are composed of a core enriched in triglycerides (TGs) and sterol esters (SEs), which are surrounded by a phospholipid monolayer due to the hydrophobicity of neutral lipids [1]. Many proteins are localized in the phospholipid membranes and are believed to function in the storage, transport, and metabolism of lipids, in signaling, and in providing a specialized microenvironment for metabolism [2]. LDs are highly dynamic organelles that are prone to changes depending on the cycles of nutrient availability; lipids are stored in LDs during nutrient-surplus conditions and are mobilized for energy production during starvation [3]. Therefore, the proteins present in LDs play unique roles in these processes. 

Despite advances in the field in recent years, the mechanisms underlying LD biogenesis remain poorly understood. It has been suggested that LDs are derived from the endoplasmic reticulum (ER) and that LD biogenesis includes neutral lipid synthesis, lens formation, and LD budding, growth, and maturation [3]. Proteins on the phospholipid membrane of LDs play a key role in LD formation. Generally, these proteins originate from the following two locations: the ER and the cytochylema [4]. Among these proteins, the enzymes that produce neutral lipids, including glycerol-3-phosphate acyltransferase 4 (GPAT4) and diacylglycerol acyltransferase 1/2 (DGAT1/2), play key roles [5]. Other proteins, such as seipin, perilipins, fat storage-inducing transmembrane (FIT), and ER-shaping proteins (reticulons and atlastin), play a role in inducing LDs [6]. However, our understanding of LD formation in fish is still lacking; particularly, the functions of many LD proteins, as well as their modulation, require further investigation and validation. 

Dehydrogenase/reductase (SDR family) member 3 (Dhrs3) is a typical member of the SDR family that was first cloned from the bovine retina [7,8]. In mammals, the overexpression of dhrs3 increases retinol ester content [9]. In zebrafish, *dhrs3* is divided into *dhrs3a* and *dhrs3b* subtypes. Knockout of *dhrs3a* upregulates the retinal acid target genes, whereas its overexpression decreases the retinal acid signaling pathway [10]. Structurally, Dhrs3 has a partially buried amphiphilic ring structure in the cytoplasmic lobules of both the LD monomolecular membrane and the ER bilayer membrane [11]. Dhrs3 is also a class of LD proteins. Deisenroth et al. reported that DHRS3 is regulated by p53 and p63 and promotes the storage of LDs in HepG2 cells [12]; the expression of DHRS3 is increased in the ER of HepG2 cells during LD formation, and the protein is mainly enriched in the monolayer phospholipid membrane produced by LDs in the ER [12]. Moreover, the role of DHRS3 in lipid deposition has been reported in other cells such as macrophages [13]. Although DHRS3 plays an important role in lipid accumulation in mammals, its role in fish has not yet been reported, and its function in controlling lipid formation is unknown. 

Hypoxia-inducible factor 1 (HIF1) is a nuclear transcription factor composed of HIF1α and HIF1β and plays a role when cells are in a hypoxic state. HIF-1 can bind to its target genes and promote their transcription to produce a series of hypoxia-adaptive responses in the body or cells [14]. HIF-1 plays an important role in hypoxia-induced lipid accumulation; a double luciferase reporter assay revealed that HIF-1 knockout, which directly regulates adipose differentiation-related protein (ADRP), can reduce fat formation in mice [15]. 

In this study, we performed a comparative transcriptome analysis of LD-containing zebrafish liver (ZFL) cells using RNA sequencing. We show that both *dhrs3a* and *dhrs3b* were upregulated in these cells and that *dhrs3a* knockdown delayed LD formation. We also show that the product of enzymatic conversion by Dhrs3, retinol, plays a role in maintaining LD formation. Moreover, Hif1α may be an important transcription factor of *dhrs3* in zebrafish. Our study provides new insights into the mechanism of LD formation in fish and suggests a new potential therapeutic target for the treatment of fatty livers in aquaculture.

## 2. Results

### 2.1. LD Accumulation Is Accompanied by Increased Dhrs3 Expression

**Figure 1:**
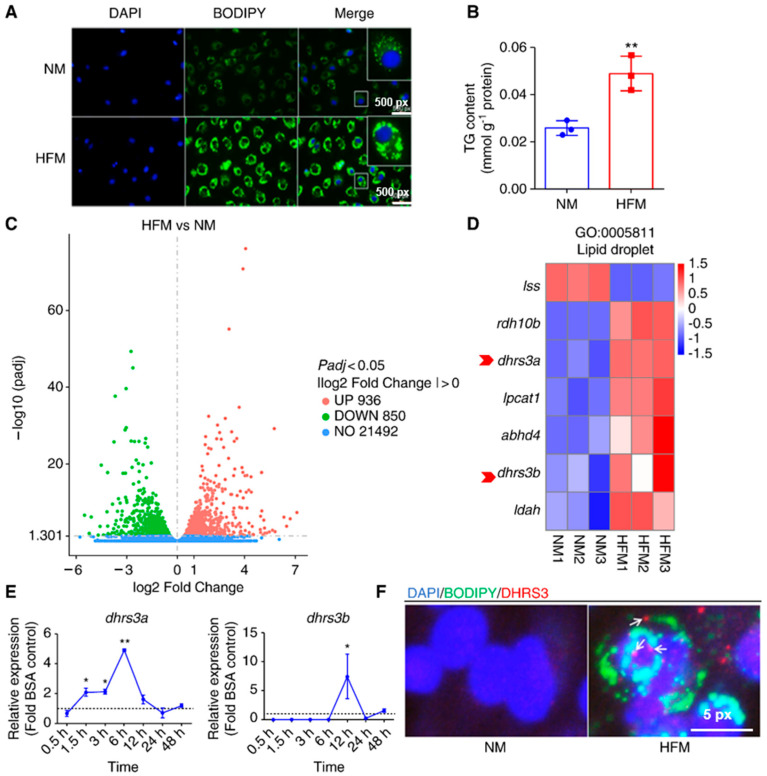
Dhrs3 expression increases in response to lipid incubation in zebrafish liver (ZFL) cells. Cells were treated with either normal medium (NM) or high-fat medium (300 μM oleic acid, HFM) for 6 h. (**A**) BODIPY (green) staining lipid droplets (LDs), and DAPI (blue) staining nuclei; images were taken using an inverted fluorescence microscope. (**B**) Total triglyceride (TG) content of cells (*n* = 3). (**C**) Volcanic plot of expressed genes; green dots indicate downregulated genes in HFM-treated cells compared to NM-treated cells, and red dots indicate upregulated genes in HFM-treated cells compared to NM-treated cells. (**D**) Differentially expressed genes enriched in GO term 0005811 (cellular component LDs), red arrows refer to the selected genes. (**E**) Relative gene expression of *dhrs3a* and *dhrs3b* at different incubation time points (*n* = 3). (**F**) LDs and nuclei were stained as previously described; Dhrs3 was stained with a specific immunofluorescent antibody (red), the arrows refer to the Dhrs3 puncta. Lss, lanosterol synthase (2,3-oxidosqualene-lanosterol cyclase); rdh10b, retinol dehydrogenase 10b; dhrs3, dehydrogenase/reductase (SDR family) member 3; lpcat1, lysophosphatidylcholine acyltransferase 1; abhd4, abhydrolase domain containing 4; ldah, LD-associated hydrolase. Statistical significance is denoted with asterisks as follows: * *p* < 0.05; ** *p* < 0.01.

To obtain the transcript profiles of ZFL cells during LD accumulation, we collected cells incubated in normal medium (NM) and high-fat medium (HFM), representing two types of fat-accumulating cells (Figure 1A,B), and subjected them to RNA sequencing. In total, 320,061,096 raw reads were generated (Table S1). Among them, 314,128,116 high-quality clean reads (47.12 G) were obtained after filtering low-quality reads from the raw data. At least 90% of the reads matched the reference genome (Supplementary Table S1). A total of 936 and 850 differentially expressed genes (DEGs) were upregulated and downregulated, respectively, in the cells exposed to HFM compared to those exposed to NM (Figure 1C; Table S2). 

In the gene ontology (GO) enrichment analysis, most upregulated DEGs were enriched in the “carboxylic acid metabolic process”, “oxoacid metabolic process”, “tRNA aminoacylation for protein translation”, and “cellular amino acid metabolic process”, whereas most downregulated DEGs were enriched in “sterol biosynthetic process”, “sterol metabolic process”, “muscle organ development”, and “steroid biosynthetic process” (Figure S1). We selected the LD-localized GO terms and identified seven DEGs; among them, *lss* was significantly downregulated, whereas *retinol dehydrogenase 10b* (*rdh10b*), *dhrs3a*, *lysophosphatidylcholine acyltransferase 1* (*lpcat1*), *abhydrolase domain containing 4* (*abhd4*), *dhrs3b*, and *LD-associated hydrolase* (*ldah*) were significantly upregulated in cells in HFM (Figure 1D). 

To verify the accuracy of the transcriptome data, the mRNA expression of *dhrs3a* and *dhrs3b* was determined using quantitative real-time reverse transcription polymerase chain reaction (qRT-PCR). The mRNA expression of *dhrs3a* was significantly increased after the cells were incubated in the lipid medium for 1.5 h, with the expression peaking at 6 h and decreasing after 12 h (Figure 1E). Notably, the mRNA expression of *dhrs3b* was only significantly increased at 12 h, and no expression was noted before 6 h (Figure 1E). To clearly visualize the expression pattern of Dhrs3, we performed immunofluorescence staining of the protein. Cells in HFM showed an increase in Dhrs3 puncta. Dhrs3 was not observed on the enveloped LDs, but some presented punctate in the cytoplasm (Figure 1F).

### 2.2. Dhrs3a Knockdown Delays LD Accumulation in Cells in a Lipid-Rich Medium 

**Figure 2:**
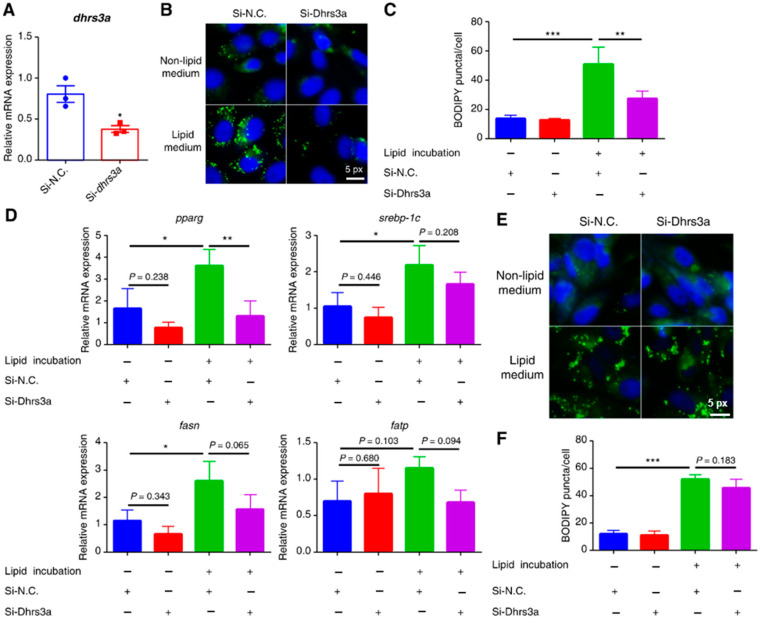
Knockdown of *dhrs3a* delays lipid droplet accumulation in response to incubation in a lipid medium. Cells were pretreated with negative control (NC), *dhrs3a* SiRNA, and incubated either with a non-lipid medium or lipid medium for 6 h (**B**–**D**) and 24 h (**E**,**F**). (**A**) Quantitative real-time reverse transcription polymerase chain reaction verification of the knockdown efficiency of *dhrs3a* using the RNAi technology (*n* = 3). (**B**,**E**) BODIPY (green) staining LDs and DAPI (blue) staining nuclei. (**C**,**F**) LD puncta per cell was measured and quantified using ImageJ 1.53q (V1.8.0.112; *n* = 3). (**D**) Quantitative reverse transcription polymerase chain reaction analysis of the relative expression of lipid metabolism-related genes (*n* = 3). pparg, peroxisome proliferator-activated receptor γ; srebp-1c, sterol regulatory element-binding protein-1c; fasn, fatty acid synthase; fatp, fatty acid transport protein. Statistical significance is denoted with asterisks as follows: * *p* < 0.05; ** *p* < 0.01; *** *p* < 0.001.

To explore the role of Dhrs3a in LD accumulation in ZFL cells, we suppressed the expression of *dhrs3a* in cells using RNAi technology and incubated these cells with lipids. The mRNA expression of *dhrs3a* decreased by 52.70% after treatment with siRNA against *dhrs3a* (Figure 2A). After incubation with the lipid-rich medium for 6 h, LD accumulation decreased in the Si-*dhrs3a*-treated cells compared to that in Si-N.C.-treated cells. However, this phenomenon was not observed in the cells incubated in the non-lipid medium (Figure 2B,C). At the transcript level, incubation with the lipids significantly increased the mRNA expression of *peroxisome proliferator-activated receptor gamma* (*pparg*), *sterol regulatory element-binding protein-1c* (*srebp-1c*), and *fatty acid synthase* (*fasn*). However, treatment with Si-*dhrs3a* decreased the mRNA expression of *pparg*, *srebp-1c*, *fasn*, and *fatty acid transport protein* (*fatp*); among them, *pparg* was significantly upregulated (Figure 2D). Notably, the reduction in LDs was almost completely reversed after incubation for 24 h (Figure 2E,F), indicating that Dhrs3a delays LD formation in response to exogenous lipids at an early stage of development.

### 2.3. Exogenous Retinyl Acetate Maintains LD Accumulation 

**Figure 3:**
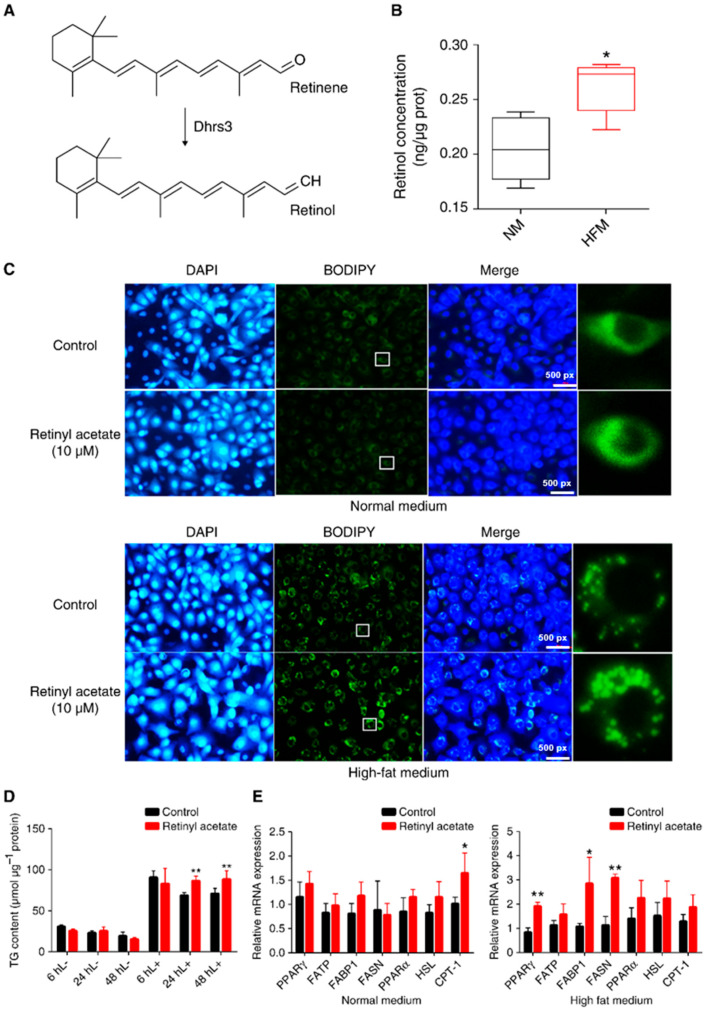
Exogenous retinyl acetate maintains LD accumulation in ZFL cells. (**A**) Schematic diagram of the function of Dhrs3. (**B**) Concentration of retinol in NM and HFM incubated cells for 24 h. (**C**) Cells were treated with or without retinyl acetate in either NM or HFM for 24 h, and the cells were stained with BODIPY (LDs, green) or DAPI (nuclei, blue). The white box delineates the magnified cells. (**D**) TG content in cells treated with or without retinyl acetate in either NM (indicated by L−) or HFM (indicated by L+) for 6, 24, and 48 h (*n* = 3). (**E**) Relative mRNA expression of lipid metabolism-related genes in cells treated with or without retinyl acetate in either NM or HFM for 24 h (*n* = 3). Statistical significance is denoted with asterisks as follows: * *p* < 0.05; ** *p* < 0.01.

The ectopic expression of Dhrs3 in cells has been reported to increase retinyl ester concentrations [9], suggesting that Dhrs3 acts as a retinal reductase to generate storage forms of retinol (Figure 3A). As expected, the retinol concentration in cells in HFM was significantly higher than that in cells in NM (Figure 3B). To explore the role of retinol in the regulation of LD accumulation, the cells were treated with or without retinyl acetate. Under NM conditions, no obvious change in LD accumulation was observed in the retinyl acetate-treated cells (Figure 3C,D). However, in the HFM, retinyl acetate-treated cells showed higher LD accumulation (Figure 3C,D). TG content was detected only in cells treated for 24 and 48 h. Notably, as time increased from 6 to 48 h, the cellular TG content decreased in the control group but did not change in the retinyl acetate group (Figure 3D), indicating that retinyl acetate maintained LD accumulation. At the molecular level, retinyl increased the expression of lipid synthesis-related genes, including *pparg*, *fatty acid-binding protein 1* (*fabp1*), and *fasn*, especially in the HFM group (Figure 3E). 

### 2.4. Exogenous Retinyl Acetate Alters the Lipidome of Zebrafish

**Figure 4:**
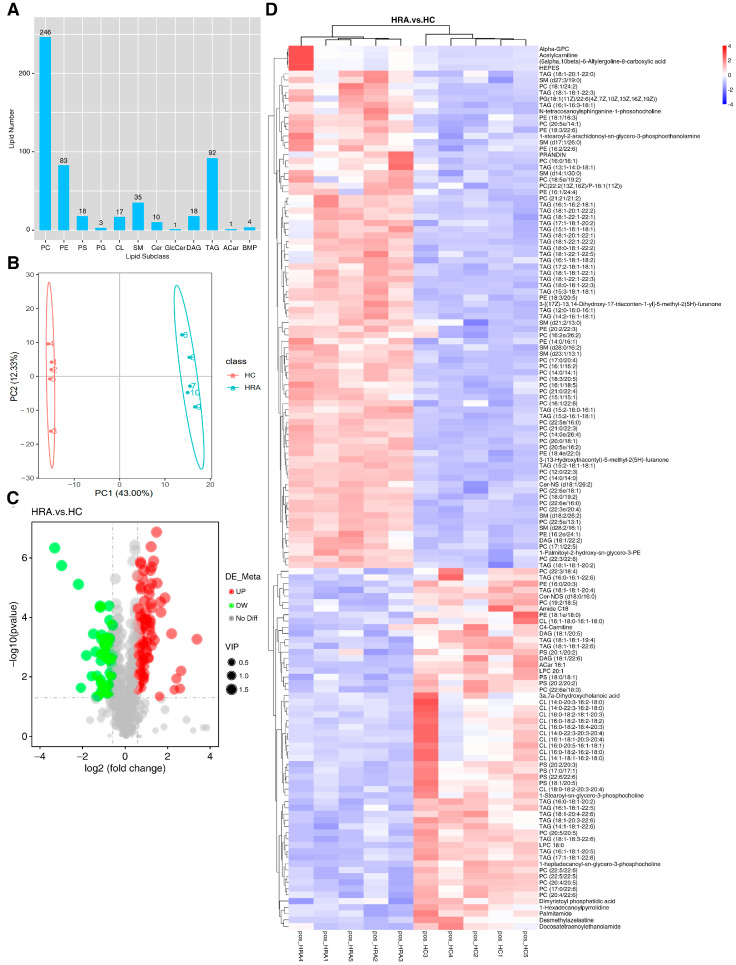
Exogenous addition of retinyl acetate alters lipid composition in ZFL cells. Cells were incubated in HFM with (HC) or without retinyl acetate (HRA) for 24 h and collected for lipidome analysis (all analyses were performed in positive ion mode; *n* = 5). (**A**) Summary of identified lipid subclasses. (**B**) Principal component analysis for the samples from different treatments. (**C**) Volcano plot of lipids. Green dots indicate low-regulated lipid compounds and red dots indicate high-regulated lipid compounds in HRA compared to HC. (**D**) Heatmap of differentially regulated lipid compounds. HFM, high-fat medium; TAG, triacylglycerols; DAG, diacylglycerols; PC, phosphatidylcholines; PE, phosphatidylethanolamines; PG, phosphatidylglycerols; PI, phosphatidylinositol; CL, cardiolipins; PS, phosphatidylserines; Cer, ceramides.

To explore the changes in lipid composition, we analyzed the lipidomes of cells treated with or without retinyl acetate in an HFM environment (Figure 4). In the positive ion mode, 246 phosphatidylcholines (PCs), 83 phosphatidylethanolamines (PEs), 92 triacylglycerols (TAGs), and 35 sphingomyelins (SMs) were identified (Figure 4A). Principal component analysis (PCA) revealed clear differences in the lipid compounds between the retinyl acetate and HFM groups (Figure 4B), and a total of 84 upregulated and 58 downregulated lipid compounds were identified in the retinyl acetate-treated cells compared with those in the HFM group (Figure 4C). The upregulated lipid compounds included 29 PCs, 25 TAGs, and 9 PEs, whereas the downregulated lipid compounds included 12 cardiolipins (CLs), 9 PCs, and 7 phosphatidylserines (PSs) (Figure 4D). No CLs or PSs were upregulated in the retinyl acetate group compared to the HFM group. In the negative ion mode, 75 PCs, 74 PEs, 34 ceramides (Cers), and 33 phosphatidylglycerols (PGs) were identified in the cells (Supplementary Figure S2A). PCA clearly separated the different treatments (Supplementary Figure S2B). A total of 28 lipid compounds were upregulated and 37 were downregulated in the retinyl acetate group compared to the HFM group (Supplementary Figure S2C). Specifically, the upregulated lipid compounds included nine PCs, eight PGs, and seven PEs, whereas the downregulated lipid compounds included 12 CLs, 8 phosphatidylinositols (PIs), and 6 PS (Supplementary Figure S2D).

### 2.5. Hif1α Regulates dhrs3a and LD Accumulation 

**Figure 5:**
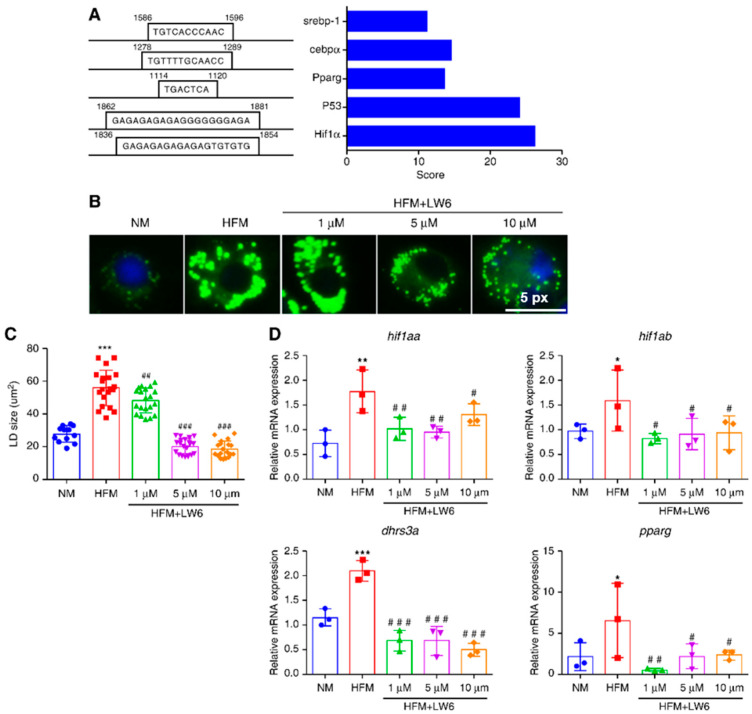
Hif1α regulates *dhrs3a* and lipid accumulation in ZFL cells. (**A**) Prediction of transcription factors for *dhrs3a* in zebrafish using an hTF target. (**B**–**D**) Cells were preincubated with or without an HIF1α inhibitor (LW6) and then transferred to HFM. Cells were stained with BODIPY (LDs, green) or DAPI (nuclei, blue). The LD size in cells was quantitated by ImageJ software (*n* = 20). Quantitative reverse transcription polymerase chain reaction analysis of the relative mRNA expression of lipid metabolism-related genes (*n* = 3). Statistical significance between HFM-treated cells and NM-treated cells was denoted with asterisks as follows: * *p* < 0.05; ** *p* < 0.01; *** *p* < 0.001; Statistical significance between HFM + LW6-treated cells and HFM-treated cells was denoted with pounds as follows: # *p* < 0.05; ## *p* < 0.01; ### *p* < 0.001.

To explore the regulation of *dhrs3a* in zebrafish, we obtained the 2000 bp promoter sequence of *dhrs3a* from NCBI and predicted its transcription factor using hTFtarget. Hif1α showed the highest prediction score among all transcriptional factors, followed by P53, Ppar-γ, CCAAT enhancer binding protein α (Cebpα), and Srebp-1 (Figure 5A). We next investigated whether Hif1α plays a role in controlling *dhrs3a* and LD accumulation. The cells were incubated with an HIF1α inhibitor, LW6, and incubated in a fatty acid-containing medium. As expected, treatment with LW6 reduced LD accumulation in response to the high fatty acid content, especially by reducing the size of LDs (Figure 5B,C). Moreover, incubation with the fatty acid-rich medium significantly increased the mRNA expression of *hif1a*, *hif1ab*, *dhrs3a*, and *pparg*, whereas the expression of these genes was suppressed by LW6 (Figure 5D). Overall, these results suggest that *dhrs3a* is regulated by Hif1α. 

## 3. Discussion

Research on the regulation of fat storage, including regulation from the perspectives of nutrition, genetic breeding, and cell culture, has become a popular topic. However, research on LD as an organelle has just begun, and the current research direction is focused on lipid catabolism [16,17,18] and adipocyte differentiation [19,20], whereas scant attention has been paid to the generation of LDs. In this study, we first showed that LD accumulation in ZFL cells is accompanied by an increase in LD protein expression levels, which plays a role in LD formation via the generation of retinol, activation of the Ppar-γ pathway, and alteration of the lipid composition. Notably, we also showed that Hif1α is one of the main transcription factors for *dhrs3a*, regulating LD formation in fish liver cells.

LD formation is a complex biological process involving different steps in which many proteins play a role [6]. In this study, we showed that only 6 h of incubation with fatty acids induced an apparent LD accumulation phenotype in cells. A total of 1786 DEGs were identified, including well-known LD biogenesis marker genes, such as *dgat* and *perilipins* (Table S2). Many of these genes are responsible for LD morphology. For example, the gene prostaglandin-endoperoxide synthase 2a, which promotes the formation of prostaglandin 2α (PGF2α), was upregulated (Table S2). Recently, we have shown that PGF2α participates in the degradation of LDs and mitochondrial development in ZFL cells, which suggests that not only were the proteins involved in LD formation induced but LD degradation was also activated [21]. In the present study, we identified seven LD proteins using GO analysis, some of which are important for modulating LD morphology. For example, LDAH is a newly identified LD protein that has recently been shown to promote LD fusion and enhance adipose triglyceride lipase (ATGL) degradation and TG accumulation [22]. LDs are surrounded by a monolayer of phospholipids, mainly PCs, which are located in the LDs, and LDAH is the key enzyme that synthesizes PCs [23]. Notably, RDH10 is a short-chain dehydrogenase essential for retinoic acid biosynthesis and has been reported to be localized to LD, allowing them to serve as sites of retinoid homeostasis [24]. Similarly, DHRS3 is involved in retinal reduction and plays an important role in retinoid metabolism [9,12]. These results suggest that retinoids are important molecules in the regulation of LD homeostasis in fish cells. 

In the present study, we focused on the protein Dhrs3, which is encoded by two gene subtypes in zebrafish: *dhrs3a* and *dhrs3b*. *Dhrs3b* did not appear to be sensitive in response to fatty acids in our study; however, it is not known whether it is more sensitive to other exogenous factors. *Dhrs3a* showed an obvious increase in response to fatty acids, and its expression subsequently decreased, which suggests that *dhrs3a* may be involved in the early stages of LD biogenesis. Indeed, our study demonstrates that *dhrs3a* knockdown can partly abolish LD accumulation after short-term incubation with a fatty acid-rich medium, but not after long-term incubation. In HepG2 cells, DHRS3 is an ER protein enriched at the focal points of LD budding, where it localizes to the phospholipid monolayer of ER-derived lipid droplets [12]. In the present study, we found that although Dhrs3 was significantly expressed in LD-containing cells, not all of the protein signals were located around LDs. These results suggest that LDs form in response to fatty acids and that Dhrs3 is involved in this process. 

It has been speculated that once retinal is reduced to retinol by DHRS3 in mammals, it can be esterified and stored with long-chain fatty acids [25]. However, this hypothesis has not yet been verified. In the present study, we showed that *dhrs3a* knockdown decreased the mRNA expression of *pparg*. Retinoid X receptor (RXR) and PPAR-γ form heterodimers that regulate the transcription of genes involved in insulin action, adipocyte differentiation, lipid metabolism, and inflammation [26]. RXR ligands include naturally occurring retinoic acid and synthetic retinoids [26]. Thus, breaking the RXR/PPAR-γ heterodimers may be one of the reasons why *dhrs3a* knockdown alters LD accumulation in cells by decreasing RXR ligands. In our study, Dhrs3 production and retinol levels were significantly increased in LD-accumulated cells. Moreover, the retinol derivative, retinyl acetate, maintained the accumulation of LDs and upregulated *pparg*, as well as its downstream genes, *fabp*, *fatp*, and *fasn*, suggesting that retinyl acetate assists in LD biogenesis in fish cells, during which the activation of the Rxr/Ppar-γ heterodimers may play a vital role. 

The structure of LDs is similar in all eukaryotic cells; it consists of a hydrophobic core formed by TAGs and steryl esters, which is surrounded by a phospholipid monolayer [27]. Moreover, this phospholipid monolayer consists of over a hundred different phospholipid molecular species, of which PC is the most abundant and is crucially important for LD stability [28,29]. Thus, it is not surprising that retinyl acetate increased the TAG and PC content in zebrafish cells in the present study, as these cells had higher LD accumulation. Moreover, we observed remarkable changes in the contents of CL, PI, and PS, which were significantly decreased in the retinyl acetate-induced LD-deposited cells. CL is a unique phospholipid that is almost exclusively located in the inner mitochondrial membrane where it is synthesized [30]. Thus, retinyl acetate may disturb mitochondrial homeostasis and lipid oxidation in cells. Moreover, changes in PI and PS may contribute to LD growth. For example, in the absence of LDs, the ER phospholipid composition is altered and displays an increase in PI content, and cells lacking seipin and Pex30 display higher levels of PI [3], suggesting that a decrease in PI may accelerate LD formation in ZFL cells. 

Studies in mammals have shown that, although *dhrs3* is regulated by P53, P63, RXR, and PPAR-γ, other transcription factors may also regulate the gene [31]. As indicated in this study, *dhrs3* is regulated by P53; however, the identification of this transcription factor is based on the fact that *dhrs3* was among the DEGs screened using microarray technology after the deletion of *p53*, which does not indicate that P53 is the only effective regulatory factor for *dhrs3* [12]. In our study, the prediction of transcription factors in the 2000 bp promoter region upstream of *dhrs3a* in zebrafish showed that the score of Hif1α was higher than that of P53, Ppar-γ, Cebpα, Srebp-1, and other traditional lipid-promoting transcription factors. We further demonstrated that inhibition of Hif1α successfully decreased the expression of *dhrs3a* and LD accumulation in ZFL cells. Although the regulation efficiency of these TFs in the transcription of *dhrs3a* is unknown, our study demonstrates the role of Hif1α in the regulation of this gene for the first time. It should be noted that the above data demonstrate a correlation between disturbance of *dhrs3a* expression and its production accompanied by altered *pparg* expression. However, the findings suggest that Ppar-γ may potentially act as a transcription factor of *dhrs3a*. It is unclear whether Ppar-γ regulates *dhrs3a* expression or if there is a reciprocal relationship between Ppar-γ and *dhrs3a* production. Although our study showed that inhibition of Hif1α decreased both *dhrs3a* and *pparg* expression, there is no evidence to suggest that Hif1α directly regulates Ppar-γ. Therefore, further research is needed to clarify the role of Ppar-γ in *dhrs3a* expression to better understand the mechanism by which Hif1α influences Dhrs3a. HIF1 is closely related to lipid metabolism and is involved in the promotion of lipid accumulation and transport, regulation of fatty acid metabolism, steroid metabolism, TG synthesis, phospholipid metabolism, and LD formation [15,32]. In current aquaculture practices, high densities and fluctuating environmental factors can result in hypoxic stress. Studies have shown increased lipid accumulation in the livers of hypoxic stress-induced fish, such as turbot and golden pompano [33,34]. Therefore, our study provides new insights into the mechanism underlying the pathogenesis of fatty liver in fish in response to hypoxia.

## 4. Materials and Methods

### 4.1. Cell Culture and Treatments

ZFL cells were cultured as previously reported [21]. Briefly, cells were cultured in a mixed medium containing 50% L-15, 35% DMEM HG, and 15% Ham’s F12 (Gibco, Thermo Fisher Scientific, Waltham, MA, USA), which was supplemented with 0.15 g/L sodium bicarbonate (Sigma-Aldrich, St. Louis, MO, USA), 15 mM HEPES (Sigma-Aldrich), 0.01 mg/mL bovine insulin (Sigma-Aldrich), 50ng/mL epidermal growth factor (Sigma-Aldrich), 5% fetal bovine serum (Gibco), and 0.5% trout serum (Caisson Labs, Smithfield, UT, USA). The cells were cultured in a cell incubator set at 28 °C. 

For the LD formation experiment, the cells were incubated either with NM or with high-fat medium (HFM) containing 300 μM bovine serum albumin (BSA)-coated oleic acid (Sigma-Aldrich) for 6 h. The BSA-coated oleic acid is obtained by adding the oleic acid dissolved in ethanol dropwise to the BSA solution to obtain a homogenous mixture. For the retinyl acetate treatment experiment, 10 μM retinyl acetate (MedChemExpress, Monmouth Junction, NJ, USA) was added to cells cultured in NM or HFM, followed by incubation for 6 h.

### 4.2. RNA Interference and Treatment with an Inhibitor

ZFL cells cultured to 75% confluence were used for RNA interference and pharmacological inhibition experiments. siRNAs were designed and synthesized using GenScript (Nanjing, China). The cells were transfected with either specific siRNA sequences against *dhrs3a* (sense: 5′-CGUUCCUCGCUCUUUCCUU-3′; antisense: 5′-GGAAAGAGCGAGGAACGUU-3′) or non-targeting siRNA (negative control; 5′-UCGCCGCUCUCUCACUUCU-3′) using a transfection reagent (Invitrogen, Thermo Fisher Scientific, Waltham, MA, USA) and incubated for 24 h. For the inhibition experiment, cells were preincubated with different concentrations of the HIF1α inhibitor, 3-[2-(4-adamantan-1-yl-phenoxy)-acetylamino]-4-hy-droxy-benzoic acid methyl ester (LW6; 1, 5, or 10 μM; MedChemExpress) for 2 h; thereafter, these cells were incubated in HFM for 6 h.

### 4.3. LD Staining

For LD staining, ZFL cells were seeded in 6-well plates at a density of 1.2 × 10^6^ cells per well and allowed to grow for 24 h, with three replicates per assay. LDs and nuclei were stained with BODIPY™ 493/503 (Invitrogen) and DAPI (Invitrogen), respectively. The methods used for fluorescence staining, image acquisition, and LD quantification were based on a previous study [21]. Briefly, the cells were fixed in 10% formalin, stained with BODIPY for 30 min and DAPI for 10 min, and imaged using an inverted fluorescence microscope with a ×40 objective (Nikon TS2, Tokyo, Japan). BODIPY puncta were quantified using ImageJ software (version 1.53q; National Institutes of Health, Bethesda, MD, USA).

### 4.4. TG and Retinol Content Assay

Cells were seeded in 6-well plates at a density of 1.2 × 10^6^ cells/well. Three replicates were used for each treatment group. After treatment, the cells were collected by trypsinization. The TG content was assayed using an enzymatic kit (Applygen, Beijing, China). The retinol content was determined using an ELISA kit (CED051Ge; Cloud-Clone Co., Houston, TX, USA). 

### 4.5. Immunofluorescence Staining

Immunofluorescence staining was performed as previously described [21]. Briefly, the cells were seeded in 6-well plates at a density of 1.2 × 10^6^ cells/well and incubated for 24 h. After treatment, the cells were fixed with 4% paraformaldehyde and incubated with Triton X-100 (0.5%; Leagene, Beijing, China). Bovine serum albumin (5%) was then added for blocking. The cells were then incubated overnight with a primary antibody against DHRS3 (ab236603; Abcam, Cambridge, UK; diluted at a concentration of 1:1000 in fetal bovine serum) at 4 °C, and subsequently with a secondary antibody conjugated to Alexa Fluor 488 (Cell Signaling Technology, Danvers, MA, USA) for 2 h. The LDs and nuclei were stained, and the cells were photographed.

### 4.6. Transcriptome Analysis

Cells were seeded in 6-well plates at a density of 1.2 × 10^6^ cells/well and treated with NM or HFM in three replicates. The cells were then collected using the RNA TRIzol reagent (Life Technologies Inc., Thermo Fisher Scientific) and sent to Novogene Biotechnology (Beijing, China) for transcriptome analysis. The methods for RNA extraction, RNA quality assessment, transcriptome library preparation, Illumina sequencing, transcriptome assembly, identification of DEGs, and annotation can be found in a previous report [35]. The DEGs were selected based on the following criteria: |log2 ratio| > 1 and *padj* < 0.05.

### 4.7. qRT-PCR 

For qRT-PCR analysis, cells were seeded in 6-well plates at a density of 1.2 × 10^6^ cells/well in three replicates. qRT-PCR was performed as described in a previous report [21]. Relative gene expression was calculated using the comparative CT method (2^−ΔΔCt^) [36,37]. We used *β-actin* as the reference gene. The sequences of the primers used for analysis are listed in Supplementary Table S3.

### 4.8. Lipidomic Analysis

Cells were inoculated in 25 cm^2^ plastic bottles at a density of 1.0 × 10^6^ cells/well in 5 replicates, this density can result in a range of cell density of 70–80%. After treatment, pre-cooled phosphate-buffered saline was used to wash the cells, and a 60% aqueous methanol solution (chromatographic grade) was added to collect the cells. The cell suspension was placed in a glass centrifuge tube with a Teflon-lined cap, precooled methanol was added, and the mixture was vortexed. Next, precooled methyl tert-butyl ether was added and the mixture was incubated at room temperature (~25 °C) in a shaker for 1 h. Mass spectroscopy-grade water was added to the mixture, and organic phases were layered; the mixture was incubated at room temperature for 10 min, and centrifuged (1000× *g*, 10 min). The upper organic phase (MTBE) was collected, and its nitrogen concentration was determined using a nitrogen-blowing apparatus. Analysis of the redissolved isopropyl alcohol was performed using liquid chromatography with a tandem mass spectrometry (LC-MS/MS) system (Thermo Fisher Scientific). An equal amount of supernatant was mixed from each processed sample to prepare a quality control sample.

The samples were injected into the Thermo Accucore C30 column (150 × 2.1 mm, 2.6 μm) at 40 °C for 20 min and at a solvent flow rate of 0.35 mL/min. Mobile phase A consisted of acetonitrile/water (6/4) + 0.1% formic acid + 10 mM ammonium acetate, and mobile phase B consisted of acetonitrile/isopropyl alcohol (1/9) + 0.1% formic acid + 10 mM ammonium acetate. The gradient elution was performed as per the following protocol: 70% A, 30% B, initial; 70% A, 30% B, 2 min; 57% A, 43% B, 5 min; 45% A, 55% B, 5.1 min; 30% A, 70% B, 11 min; 1% A, 99% B, 16 min; 70% A, 30% B, 18.1 min; and 70% A, 30% B, 20 min. Q Exactive^TM^ HF mass spectrometer was operated in positive [negative] polarity mode with the following parameters: sheath gas, 20 arbitrary units; sweep gas, 1 arbitrary unit; auxiliary gas rate, 5 (anion was set to 7); spray voltage, 3 kV; capillary temperature, 350 °C; heater temperature, 400 °C; S-Lens RF level, 50; scan range, 114–1700 *m*/*z*; automatic gain control target, 1e6; normalized collision energy, 25, 30 [anion was set to 20, 24, 28]; injection time, 100 ms; isolation window, 1 *m*/*z*; automatic gain control target (MS2), 1e5; dynamic exclusion: 15 s.

Raw data generated using ultra-high-performance LC-MS/MS were imported into Compound Discoverer 3.01 (CD3.1; Thermo Fisher Scientific). For accurate identification, peak alignment was performed for different samples according to a retention time deviation of 0.2 min and mass deviation of 5 ppm, and the peak area was quantified. The molecular formula was predicted based on molecular ion peaks and fragment ions and compared with the Lipidmaps, Lipidblast, and HMDB databases. The statistical software R (R version r-3.4.3), Python (Python 2.7.6), and CentOS (CentOS release 6.6) were used for the statistical analyses. Blank samples were used to remove background ions, quantitative results were normalized, and the lipid data were qualitatively and quantitatively analyzed.

PCA was performed using MetaX, a flexible and comprehensive metabolomic data processing software. The differentially regulated lipid compounds were set based on the following criteria: VIP > 1, *p* < 0.05, fold change |FC| ≥ 2, or ≤0.5. The metabolites of interest were screened using volcanic maps with Log2(FC) and −log10(*p*-value) of the metabolite content. The clustering heatmap was normalized by z-scores in the differential metabolite intensity region and drawn using the Pheatmap package in R. 

### 4.9. Statistical Analysis

All data are expressed as mean ± SD (standard deviation). Differences between NM- and HFM-treated cells or control and retinyl acetate-treated cells were determined using an independent sample *t*-test. Two-way analysis of variance (ANOVA), followed by Bonferroni’s post hoc test, was used to compare differences between experimental treatments. All analyses were performed using the PASW Statistics 18 software (SPSS, Chicago, IL, USA).

## 5. Conclusions

In the present study, we propose a new mechanism for lipid formation in fish cells in which Dhrs3a is the main LD protein that acts on LD growth at a relatively early stage. The metabolite, retinol, may contribute to this process by activating Ppar-γ and decreasing the lipid classes CL, PI, and PS. We also show that Hif1α is one of the main transcription factors regulating *dhrs3a.* Further studies should focus on the comparison of Hif1α and other TFs in the regulation of *dhrs3a* and on validating this LD formation pathway in vivo.

## Data Availability

The data described in this manuscript are all contained within the manuscript.

## Supplementary Materials

The supporting information can be downloaded at: https://www.mdpi.com/article/10.3390/ijms241210236/s1.
