# Supplementary material for: Hif1α/Dhrs3a Pathway Participates in Lipid Droplet Accumulation via Retinol and Ppar-γ in Fish Hepatocytes
Source: Int J Mol Sci. 2023 Jun 16;24(12):10236. doi: 10.3390/ijms241210236 (PMC10299640; doi:10.3390/ijms241210236)
Supplement: Supplementary file 1 [file ijms-24-10236-s001.zip › ijms-2422457-supplementary.pdf]

Supplementary Materials

Table S1. Summary of the transcriptome of zebrafish liver cells.

	NM1	NM2	NM3	HFM1	HFM2	HFM3
**Raw reads**	46,009,916	50,485,182	50,427,782	63,491,932	47,293,998	62,352,286
**Clean reads**	45,064,242	49,710,858	49,525,936	62,315,276	46,219,892	61,291,912
**Clean bases (bp)**	6.76G	7.46G	7.43G	9.35G	6.93G	9.19G
**Error rate (%)**	0.03	0.03	0.03	0.03	0.03	0.03
**Q20 (%)**	96.91	97.99	96.91	96.93	96.91	97.07
**Q30 (%)**	91.78	94.09	91.84	91.89	91.79	92.14
**GC pct (%)**	48.67	48.6	49.42	48.25	48.47	49.9
**Total map**	41,423,121 (91.92%)	46,183,299 (92.9%)	45,614,072 (92.1%)	57,063,807 (91.57%)	42,428,147 (91.8%)	56,935,905 (92.89%)

Table S2. Summary of differentially expressed genes in zebrafish liver cells incubated in a high-fat medium (HFM) or a normal medium (NM).

See the Excel file.

Table S3. Primers used for real-time quantitative PCR.

Target gene	Forward (5′-3′)	Reverse (5′-3′)	Accession number
β-actin	TCTGGTGATGGTGTGACCCA	GGTGAAGCTGTAGCCACGCT	NM_131031.2
Dehydrogenase/reductase (SDR family) member 3a (***dhrs3a***)	GCCATCGACTATTGCACGTC	GTAGTGCATCCCACTCCAGG	NM_001003477.1
Dehydrogenase/reductase (SDR family) member 3b (***dhrs3b***)	CATGGGGACAGAGTGCCATT	TGTCCAGCAAACTCTTGCCA	NM_001006070.1
Peroxisome proliferator-activated receptor gamma (***pparg***)	CAACTGCAGATACATGCCGC	TGGTAGCTGTGGAAGAAGCG	NM_131467.1
Sterol regulatory element binding transcription factor 1 -C (***srebp1-c***)	CAGAGGGTGGGCATGCTGGC	ATGTGACGGTGGTGCCGCTG	NM_001105129.1
Fatty acid synthase isoform X2 (***fasn***)	AAGCTTTCCGCTACATGGCT	TTGACTCCTCTGAACGCACC	XM_009306806.3
Fatty acids transporter (***fatp***)	CGTGTGGCTTCAACAGTGTG	ATAGTGTCCTCGTCTGCCCT	NM_001077248.1
Fatty acid binding protein 1a (*fabp1a*)	GTGTGCCGGATGATGAGGTC	TGACTTTAGTTCCGGCCGTC	NM_001044712.1
Peroxisome proliferator-activated receptor alpha (***ppara***)	TCAGCGGGAAAGAGGAACAC	GAAACACACGTGCTTTGGCT	NM_001161333.1
Hormone-sensitive lipase (***hsl***)	GCCCAAGGAGCAACAAACTG	TGCAGAGGCTGTTGATGAGG	NM_001316725.1
Carnitine palmitoyltransferase 1 (***cpt-1***)	TCTACCTGAGAGGTCGTGGG	TGACGTTTCCTGCTCTTGCT	NM_001044854.1
Hypoxia-inducible factor 1 subunit alpha a (***hif1aa***)	GGGAGTGGGTTTGGATCTGG	GACTGGAGAAACAGTCCGCA	NM_001308559.1
Hypoxia-inducible factor 1 subunit alpha b (***hif1ab***)	ACCCCAAGAATTCTCAGCCG	GGACAGGACTACATCCCCCT	NM_001310042.1

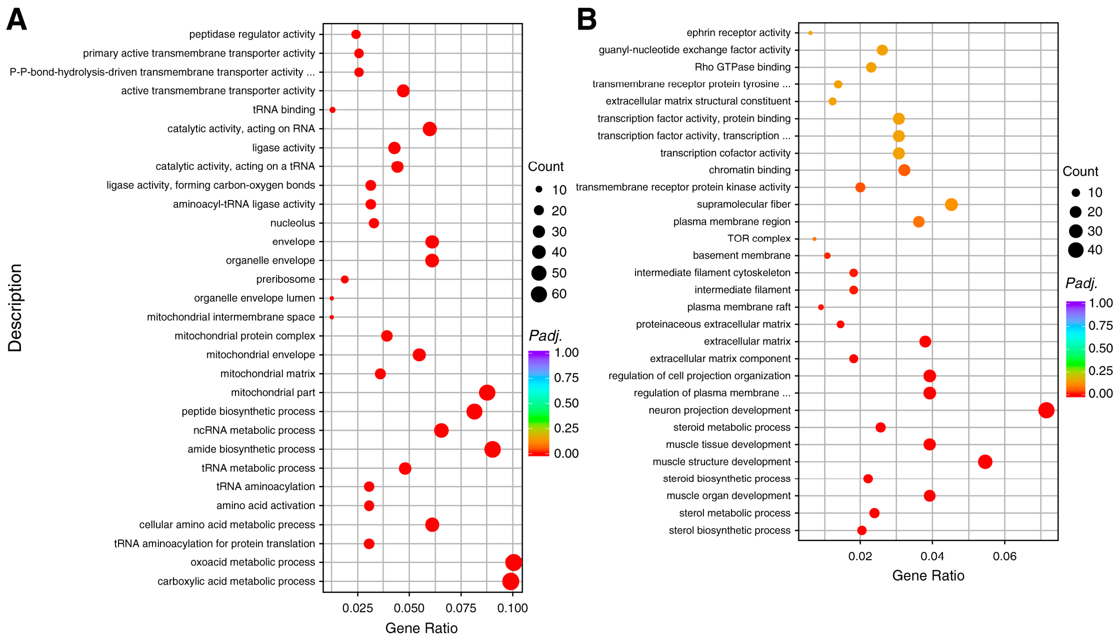

Figure S1. Gene ontology (GO) categories of the upregulated (A) and downregulated (B) differentially expressed genes in zebrafish liver (ZFL) cells in response TO culture in either normal medium (NM) or high-fat medium (HFM) for 6 h.

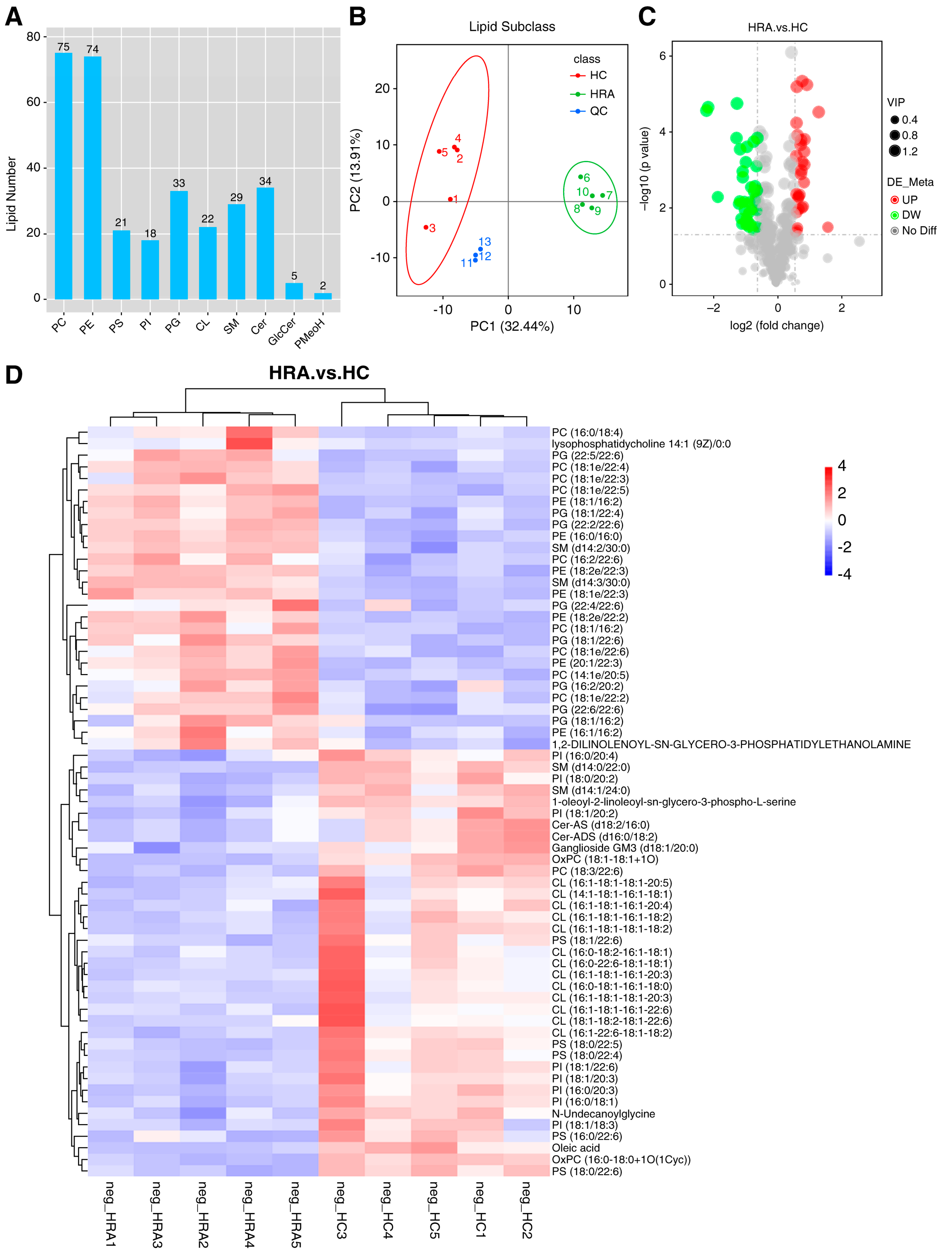

Figure S2. Exogenous retinyl acetate alters lipid composition in zebrafish liver (ZFL) cells. Cells were incubated in a high-fat medium with (HC) or without retinyl acetate (HRA) for 24 h and collected for lipidome analysis (all analyses were performed in the negative ion mode; *n* = 5). A, Summary of identified lipid subclasses. B, Principal component analysis for the samples from different treatments. C, Volcanic plot of lipids; green dots indicate low-regulated lipid compounds and red dots indicate high-regulated lipid compounds in HRA compared with those in HC. D, Heatmap of differentially regulated lipid compounds. TAG, triacylglycerol; DAG, diacylglycerol; PC, phosphatidylcholines; PE, phosphatidylethanolamines; PG, phosphatidylglycerols; PI, phosphatidylinositol; CL, cardiolipin; PS, phosphatidylserine; Cer, ceramide.
